# Fast Growth May Impair Regeneration Capacity in the Branching Coral *Acropora muricata*

**DOI:** 10.1371/journal.pone.0072618

**Published:** 2013-08-30

**Authors:** Vianney Denis, Mireille M. M. Guillaume, Madeleine Goutx, Stéphane de Palmas, Julien Debreuil, Andrew C. Baker, Roxane K. Boonstra, J. Henrich Bruggemann

**Affiliations:** 1 Laboratoire d’Ecologie Marine – FRE 3560 CNRS, Université de La Réunion, Saint-Denis, La Réunion, France; 2 Département Milieux et Peuplements Aquatiques, UMR CNRS-MNHN-UPMC-IRD BOrEA, Muséum National d’Histoire Naturelle, Paris, France; 3 Laboratoire d’Excellence ‘CORAIL,’ Perpignan, France; 4 Aix-Marseille Université, CNRS/INSU, IRD, Mediterranean Institute of Oceanography (MIO), UM 110, Marseille, France; 5 Université de Toulon, CNRS/INSU, IRD, Mediterranean Institute of Oceanography (MIO), UM 110, La Garde, France; 6 Division of Marine Biology and Fisheries, Rosenstiel School of Marine and Atmospheric Science, University of Miami, Miami, Florida, United States of America; California Polytechnic State University, United States of America

## Abstract

Regeneration of artificially induced lesions was monitored in nubbins of the branching coral *Acropora muricata* at two reef-flat sites representing contrasting environments at Réunion Island (21°07′S, 55°32′E). Growth of these injured nubbins was examined in parallel, and compared to controls. Biochemical compositions of the holobiont and the zooxanthellae density were determined at the onset of the experiment, and the photosynthetic efficiency (*F_v_/F_m_*) of zooxanthellae was monitored during the experiment. *Acropora muricata* rapidly regenerated small lesions, but regeneration rates significantly differed between sites. At the sheltered site characterized by high temperatures, temperature variations, and irradiance levels, regeneration took 192 days on average. At the exposed site, characterized by steadier temperatures and lower irradiation, nubbins demonstrated fast lesion repair (81 days), slower growth, lower zooxanthellae density, chlorophyll *a* concentration and lipid content than at the former site. A trade-off between growth and regeneration rates was evident here. High growth rates seem to impair regeneration capacity. We show that environmental conditions conducive to high zooxanthellae densities in corals are related to fast skeletal growth but also to reduced lesion regeneration rates. We hypothesize that a lowered regenerative capacity may be related to limited availability of energetic and cellular resources, consequences of coral holobionts operating at high levels of photosynthesis and associated growth.

## Introduction

Disturbances from multiple biotic and abiotic factors cause recurring losses of living tissues from coral colonies [1] and may result in sporadic severe reductions of living corals at the reef scale [2]. A colony’s integrity is maintained through rapid tissue repair [3], while regrowth from surviving colony parts greatly accelerates the recovery of damaged reefs [4] and may even reverse coral-algal phase shifts [5]. Thus, the growth and regenerative capacities of corals are fundamental to determining the resilience of reefs [5], [6] and are often used as indicators of coral colony condition and generalized to define a reef’s health status [7], [8].

Coral colony growth and regeneration are likely closely related processes, involving calcification and tissue extension [9]. Repair of damaged colony parts (hereafter defined as ‘lesions’) requires energy [10] and interstitial cells [11]–[13] and competes for these limited resources with other essential biological processes, such as growth [14], reproduction [15], disease resistance, and competitive ability [16]. Lesion regeneration is facilitated by the clonal architecture of corals, enabling the transport and reallocation of resources among units [10], [11], to which polyps proximal to the damaged area contribute the most [14], [17]. Lesion regenerative capacity varies with the lesion size and shape [18]–[20], colony morphology and species (see review in [21]), and environmental conditions [8], [22]. Also, coral colony growth is known to vary with these parameters (e.g. in [23]–[25]). While trade-offs between colony growth and lesion repair were documented at the level of individual colonies [17], high skeletal extension rates are generally associated with an efficient lesion-repair capacity (see review in [21]). Hence, fast-growing branching corals have higher lesion regeneration rates with full recovery [26] compared to more-slowly growing massive corals [17].

The present study was designed to investigate the lesion regenerative capacity of the branching coral *Acropora muricata* at Réunion Island in contrasting natural environments. An earlier study on this species revealed a trade-off between regeneration and growth, and indicated that they were independent of size [27]. We further investigated relationships between skeletal growth and lesion regeneration by monitoring both processes in experimental coral nubbins on two reef flats, Planch’Alizé and Kiosque, selected for their contrasting environmental conditions that induced strong differences in coral growth rates. We not only show that coral growth is inversely related to the rate of lesion repair, but also that high coral growth rates may harm regeneration capacity in *A. muricata*. We suggest that rapid coral growth, promoted by a high zooxanthellae density and high photosynthetic efficiency, compromised their capacity to invest energy and cellular resources in lesion repair. We hypothesize that maintaining coral-symbiont functioning under conditions that boost zooxanthellae densities and their photosynthetic rate implies their sequestering of an important share of host resources. This may particularly involve the use of the energy-rich photosynthetic compounds produced by the *Symbiodinium*.

## Methods

### Ethics Statement

This experiment was conducted and corals were sampled with permission granted by the Réserve Naturelle Marine de la Réunion. All survey procedures carried out were done with proper precautions to minimize impacts to the reefs.

### Study Area and Species

Experiments were conducted on the west coast of Réunion Island (21°07′S, 55°32′E) at two shallow (1∼2 m deep) reef-flat sites 11 km apart from each other: Planch’Alizé and Kiosque. At each site, the seawater surface temperature (SST) was measured at hourly intervals using calibrated underwater temperature loggers (Hobo Water Temp Pro, with an accuracy of 0.2°C, Onset Computer Corp., USA). Solar radiation (J cm^−2^) data were obtained from the French Meteorological Service on land at station close to each site (stations no. 97415590 and 97413545).

**Figure 1:**
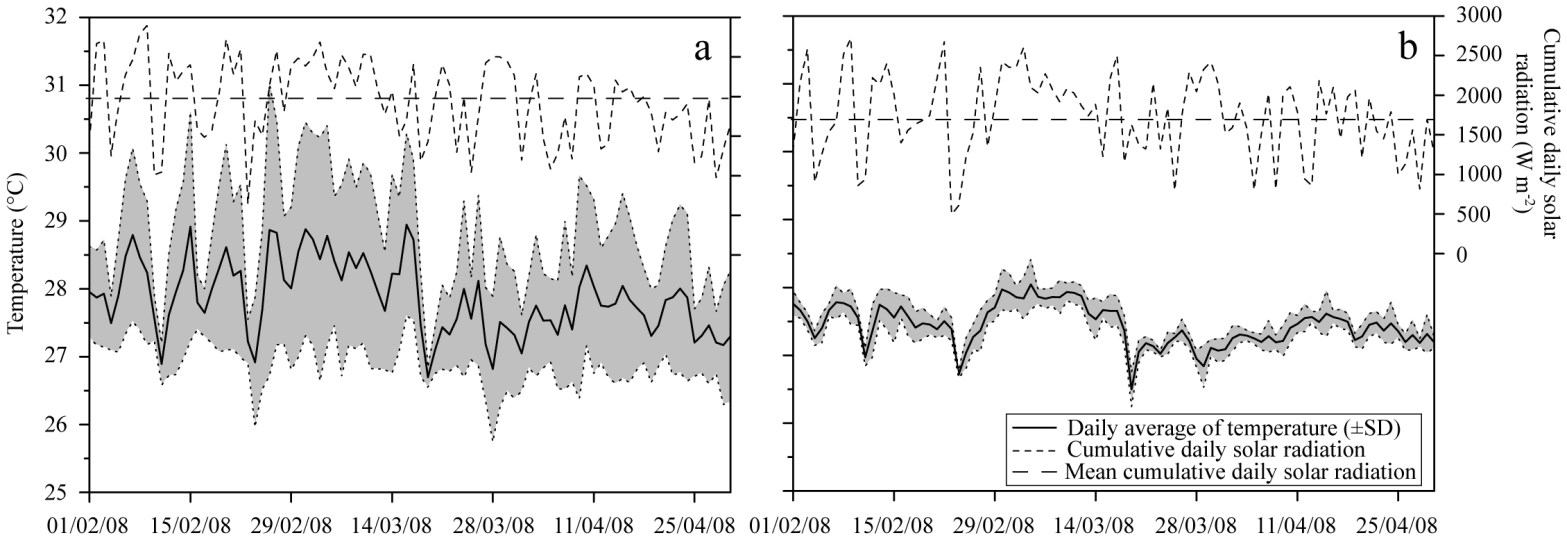
Environmental conditions. Daily average sea surface temperature (SST; ± SD, gray area) and cumulative daily solar radiation during the experimental period at (**a**) Planch’Alizé and (**b**) Kiosque.

Situated at the la Saline reef, Planch’Alizé is a sheltered site, located downstream of seawater flowing over the 500-m-wide reef flat. Low water flow and high solar radiation contribute to heating the reef water during the day, inducing important daily SST variations (Fig. 1a) and higher average SSTs compared to the second site. In contrast, Kiosque is an exposed site located at the Saint-Leu reef; waves impinging on the narrow reef flat (200 m wide) induce strong water motion and an influx of coastal water that reduces daily SST fluctuations (Fig. 1b). Moreover, solar radiation at this site is often tempered due to cloud formation along the steep slopes above the Saint-Leu reef, especially during the hot season. Further details of environmental conditions at these study sites can be found in [22].

The branching coral *A. muricata* (Linnaeus, 1758), senior synonym of *A. formosa* [28], is a dominant species of Réunion reef-flat coral communities and a common species in the Indo-Pacific region [28]. At Planch’Alizé, this species often co-occurs with *Montipora circumvallata* and *Porites (Synaraea) rus*, forming large thickets. At this site, benthic communities comprise a high proportion of dead corals covered by algal turfs (∼30% in 2008, data from GCRMN monitoring). This site is further characterized by nutrient enrichment originating from N-rich groundwater seepage near the shore [29]. At Kiosque, coral diversity is higher, but the density of *A. muricata* is lower than at Planch’Alizé [30], [31].

### Sample Preparation and Monitoring of Lesion Regeneration and Growth

At each site, 15 nubbins (7 cm long) were sampled from each of 5 haphazardly selected *A. muricata* colonies separated by >10 m in distance. Nubbins were glued onto numbered acrylic glass tiles, mounted on racks positioned in a representative area at each native reef flat site, and left to recover for 2 months prior to the experiments. On March 8, 2008, 5 replicate nubbins from each colony were randomly selected, and artificial circular lesions of 11.5±0.6 mm in diameter and 3 mm in depth were inflicted using a grinding stone at mid-height on the side of each branch, removing all traces of living tissue. Particular care was taken to induce lesions of a constant size and depth. Nubbins were then left to heal at their respective native sites.

The surface area of lesions and projected vertical surface area of nubbins were monitored until April 30, 2008 and quantified with CPCe software [32] from digital photographs taken with a camera mounted on a support (in order to maintain a constant distance to the sample) equipped with a scale. The monitoring frequency was adjusted to the rate of regeneration.

The relative mass increase (‰ d^−1^) of nubbins was calculated from the difference between the initial and final projected nubbin surface area multiplied by the mean site-specific skeletal density, divided by the duration of the experiment. The calcification rate, or mass of CaCO_3_ deposited per unit area per day (g cm^−2^ d^−1^), was estimated for each nubbin as the product of its linear extension and skeletal density. The skeletal density (g cm^−3^) of nubbins was calculated as the dry weight-to-volume ratio, following Bucher et al. [33]. The volume was determined by dipping nubbins in molten paraffin wax to form a water-tight barrier and determining their buoyant mass in distilled water at 20°C.

The remaining undamaged nubbins (9 or 10 per colony) were used as controls, and their growth (relative increases in the buoyant mass and surface area) was monitored simultaneously.

### Photosynthetic Efficiency, *Symbiodinium* Identification, Density of Zooxanthellae, and Biochemical Composition of Holobionts

The photosynthetic efficiency of nubbins was quantified using the maximum dark-adapted quantum yield, *F_v_/F_m_* (*F_v_* is the difference between *F_0_*, the initial fluorescence, and *F_m_*, the maximum fluorescence [34]). Measurements were made with a diving-PAM (Walz, Germany) at night, 1 h after sunset in order to maximize the frequency of open photosystem II reaction centres, using a custom-built nubbin holder to ensure a constant probe distance and measurement location. Following diving-PAM settings were used along the experiment: saturating intensity = 8, saturating width = 0.6, gain = 4, damping = 2. On injured nubbins, *F_v_/F_m_* was assessed at each monitoring time, about 2 cm away from the lesion borders. *F_v_/F_m_* of control nubbins was determined at the beginning, half-way, and end of the experiment.

For 3 of the 5 colonies per site, 1 undamaged nubbin was sampled before the beginning of the experiment (February 28, 2008), snap frozen in liquid nitrogen and stored at −80°C. Genomic DNA was then extracted from a subsample using a Qiagen® Blood and Tissue Kit (Santa Clarita, CA, USA). The internal transcribed spacer (ITS)-2 region of *Symbiodinium* ribosomal (r) DNA was amplified using the primers “ITSintfor2” and “ITS2revclamp” [35] under the following conditions: an initial denaturing step of 94°C for 3 min followed by 35 cycles of 1 min at 94°C, 1 min at 58°C, and 1 min at 74°C, followed by a single cycle of 7 min at 74°C. Amplified DNA was then analyzed by denaturing gradient gel electrophoresis (DGGE; CBS Scientific, Del Mar, CA, USA) using a denaturant gradient of 35% to 75%. Prominent bands characterizing different profiles were excised, re-amplified, and sequenced as described by LaJeunesse [36]. Sequences were identified using BLAST searches of GenBank, and exact matches were reported using the nomenclature established by LaJeunesse [35].

Tissues were removed from the skeleton using a dental jet (Waterpik Technologies, Fort Collins, CO, US) with recycled freshly filtered seawater [37], and the obtained coral blastate was homogenized with a potter homogenizer (5 min, 2000 rpm). Zooxanthellae densities were determined from an aliquot of the homogenate by first separating zooxanthellae from host tissue by centrifugation, suspension, and homogenization of the pellet in 2 mL of a formalin solution (5%). Zooxanthellae were then counted in 5 subsamples (0.2 mm^3^ each) using a hemocytometer at 400×magnification. The chlorophyll (Chl) *a* concentration was determined by spectrometric absorbance at 664 nm [38]. The protein concentration was determined from 0.1 mL of homogenate following a modified protocol [39] of the Lowry method [40], using the Folin reagent with phenol and bovine serum albumin as standards. Finally, total lipids were extracted using a monophasic solvent mixture (CH_2_Cl_2_: CH_3_OH: H_2_O; 1∶2: 0.8 v/v/v) [41] with 100 µL of an internal standard (hexadecanone, GC grade, Sigma Chemical, St. Louis, MO, USA) during 12 h at 4°C. CH_2_Cl_2_ and H_2_O (1∶1 v/v) were added to the supernatant to create a biphasic mixture. The organic (dichloromethane) phase was collected in glass bottles with Teflon caps, evaporated under nitrogen, and stored under a nitrogen atmosphere at −20°C until being analyzed. The total lipid content was then determined using thin-layer chromatography coupled with flame-ionization detection (TLC-FID) on an Iatroscan apparatus model MK6-s (with hydrogen flow of 160 mL min^−1^ and air flow of 2000 mL min^−1^) and an i-Chromstar 6.1 integration system (SCPA, Bremen, Germany).

### Statistical Analysis

Regeneration of each nubbin was quantified by fitting the remaining lesion size (%) over time using a least-squared regression and an exponential decay model, allowing full recovery [20]. We used the following formula:
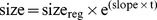
where size is the remaining lesion size (%), size_reg_ is the maximum area that can be fully regenerated, slope is the regression slope, and t is the time in days. Since there is no natural logarithm for 0 (when a lesion is completely healed), +1 was added to the remaining lesion size for the calculations.

Conformity with parametric assumptions was visually assessed from residual plots. A potential site difference in the initial lesion size was tested using a 1-way analysis of variance (ANOVA). Possible effects of the initial lesion size on the regression slopes were analyzed using an analysis of covariance (ANCOVA) with the initial lesion size as the co-variable. A site difference in model slopes was investigated using an ANOVA, with “colony” as the random factor. For each site, mean slopes were used to simulate regeneration during 300 days. The difference in relative growth between sites and treatments was compared using a 2-way ANOVA, and Tukey’s honest significant difference (HSD) test was used for post-hoc comparisons.

Welch’s *t*-test was used to compare biochemical properties of nubbins between sites at the onset of the lesion regeneration experiment. As a parametric assumption could not be met, non-parametric Mann-Whitney U-test was used to compare *F_v_/F_m_* of injured and control nubbins at the beginning, middle, and end of the experiment, and was also used to assess site differences in *F_v_/F_m_* of injured nubbins. Friedman’s ANOVA was used to test temporal variations in *F_v_/F_m_*, where values obtained from the same nubbin over time were considered dependent. Results are presented as the mean ± standard deviation (SD).

## Results

*Acropora muricata* nubbins were consistently associated with C3 *Symbiodinium*. Corals from the sheltered site Planch’Alizé were characterized by a higher zooxanthellae density, Chl *a* concentration, and total lipid content, but also not significant lower tissue biomass and protein content compared to those from the exposed site Kiosque (Table 1).

**Table 1:** Biochemical properties of nubbins of *Acropora muricata* at the onset of the lesion regeneration experiment.

Parameter	Planch’Alizé	Kiosque
Tissue biomass (mg dry weight cm^−2^)	3.27 (±0.49)	4.13 (±0.83)
Zooxanthellae density (10^6^ cells cm^−2^)*	2.11 (±0.08)	1.85 (±0.60)
Chl *a* concentration (µg cm^−2^)**	9.68 (±1.88)	8.74 (±2.09)
Protein content (mg cm^−2^)	1.99 (±0.19)	2.30 (±0.34)
Total lipids in holobiont (mg C cm^−2^)*	0.81 (±0.26)	0.51 (±0.10)

* *p*<0.05;

** *p*<0.01;

*** *p*<0.001.

**Figure 2:**
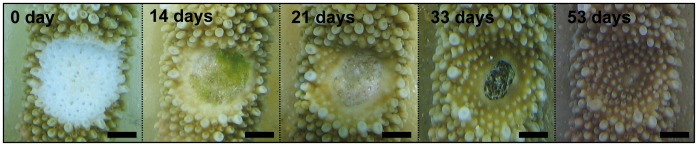
Acropora muricata. Lesion regeneration pattern at Kiosque, almost completely healed after 53 days. Scale bar = 5 mm.

**Figure 3:**
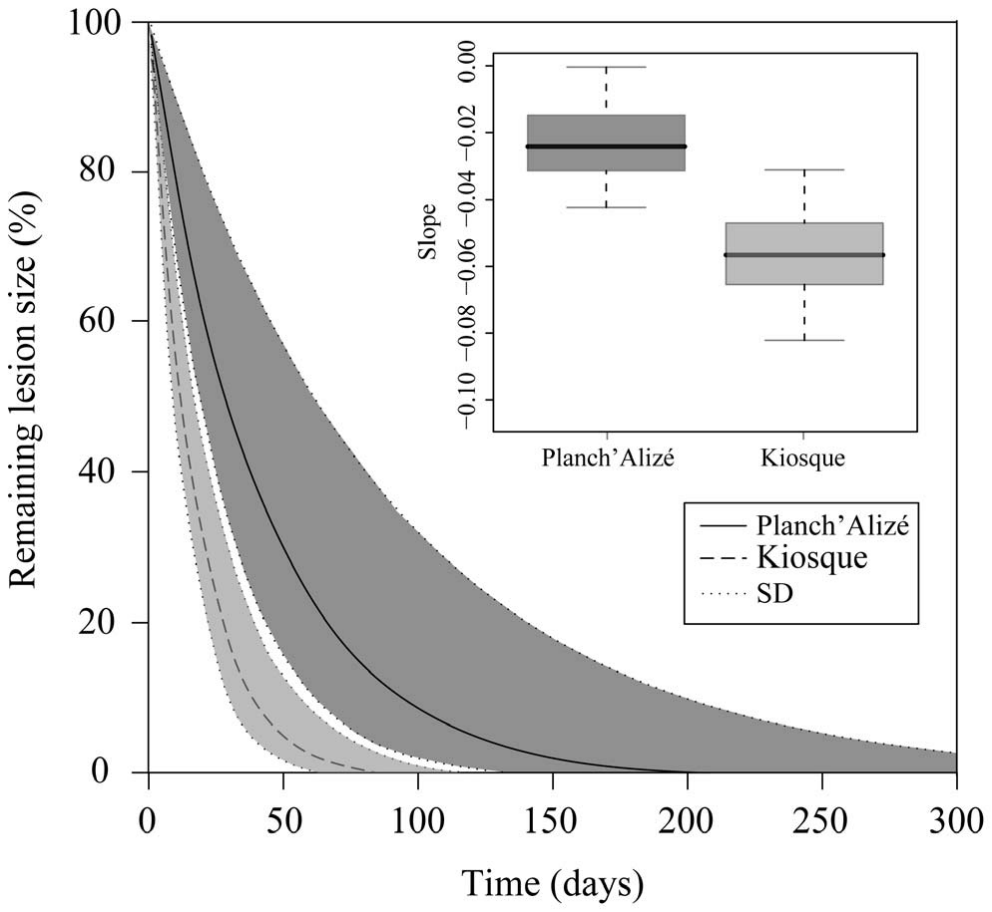
Lesion regeneration of *Acropora muricata*. Predicted size of artificial lesions over time in nubbins at Planch’Alizé and Kiosque according to slopes estimated from the regeneration model (inset).

**Figure 4:**
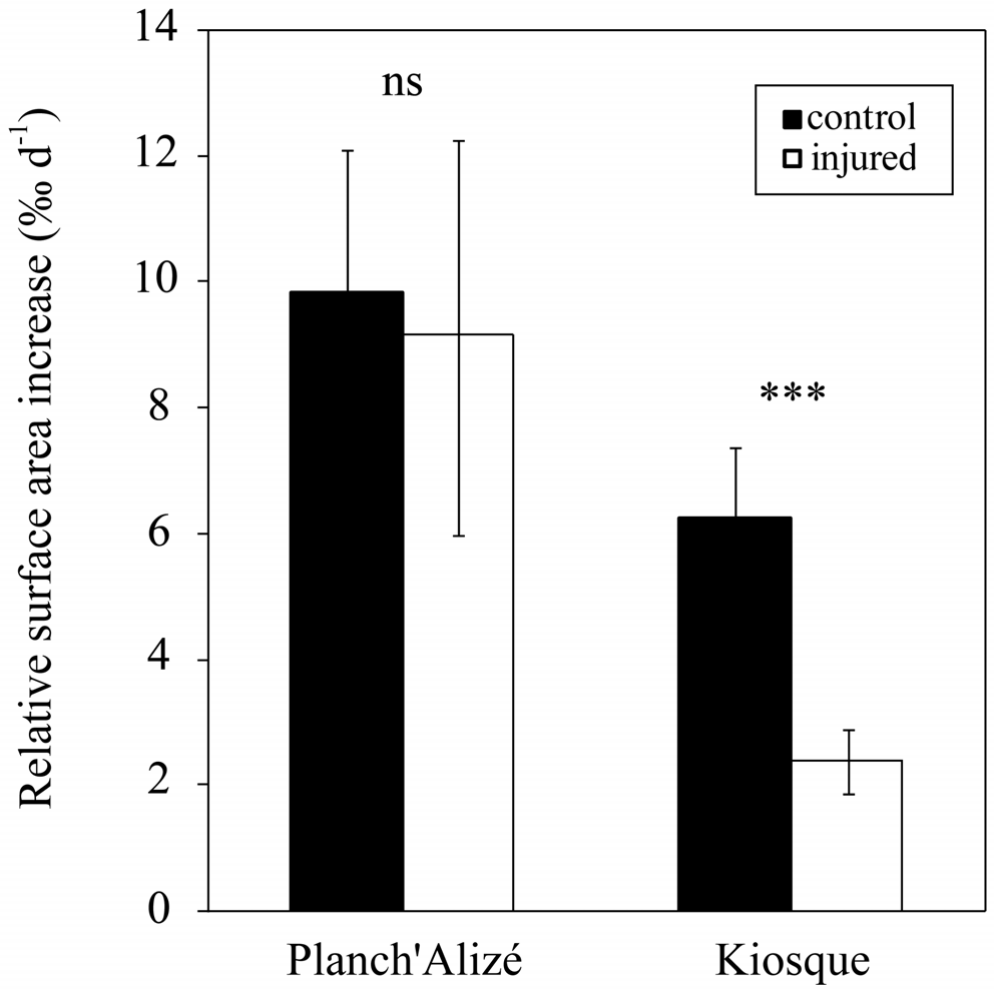
Growth of *Acropora muricata*. The mean (± SD) relative increase in the projected surface area (‰ d^−1^) of control and injured nubbins by site.

The initial lesion size was 104±11 mm^2^ (*n* = 48) and did not differ between sites (*F*_1,47_ = 2.21, *p*>0.05) or colonies (*F*_9,39_ = 1.98, *p*>0.05). Two nubbins at Kiosque died before the onset of regeneration monitoring; these were not included in the analysis. There was no mortality of nubbins during the experimental period. The lesion size decreased rapidly over time (Fig. 2). After 53 days, lesions in nubbins at Kiosque were at 3% of their initial size, while those in nubbins at Planch’Alizé were still at 24% of their initial size. Based on the outcomes of the exponential decay model, lesions healed completely after on average 81 days at Kiosque and 192 days at Planch’Alizé (Fig. 3). Regression slopes significantly differed between Planch’Alizé (−0.024±0.012, *n* = 25) and Kiosque (−0.057±0.017, *n* = 23; *F*_1,8_ = 32.89, *p*<0.001, inset in Fig. 3), and the difference was not related to colony (*F*_8,38_ = 2.17, *p*>0.05) or initial lesion size (*F*_1,43_ = 1.79, *p*>0.05). Increases in the relative surface area of nubbins (Fig. 4) varied with site (*F*_1,142_ = 121.04, *p*<0.001) and treatment (*F*_1,142_ = 25.10, *p*<0.001), with a significant interaction term observed between the two factors (*F*_1,142_ = 7.15, *p*<0.01).

**Figure 5:**
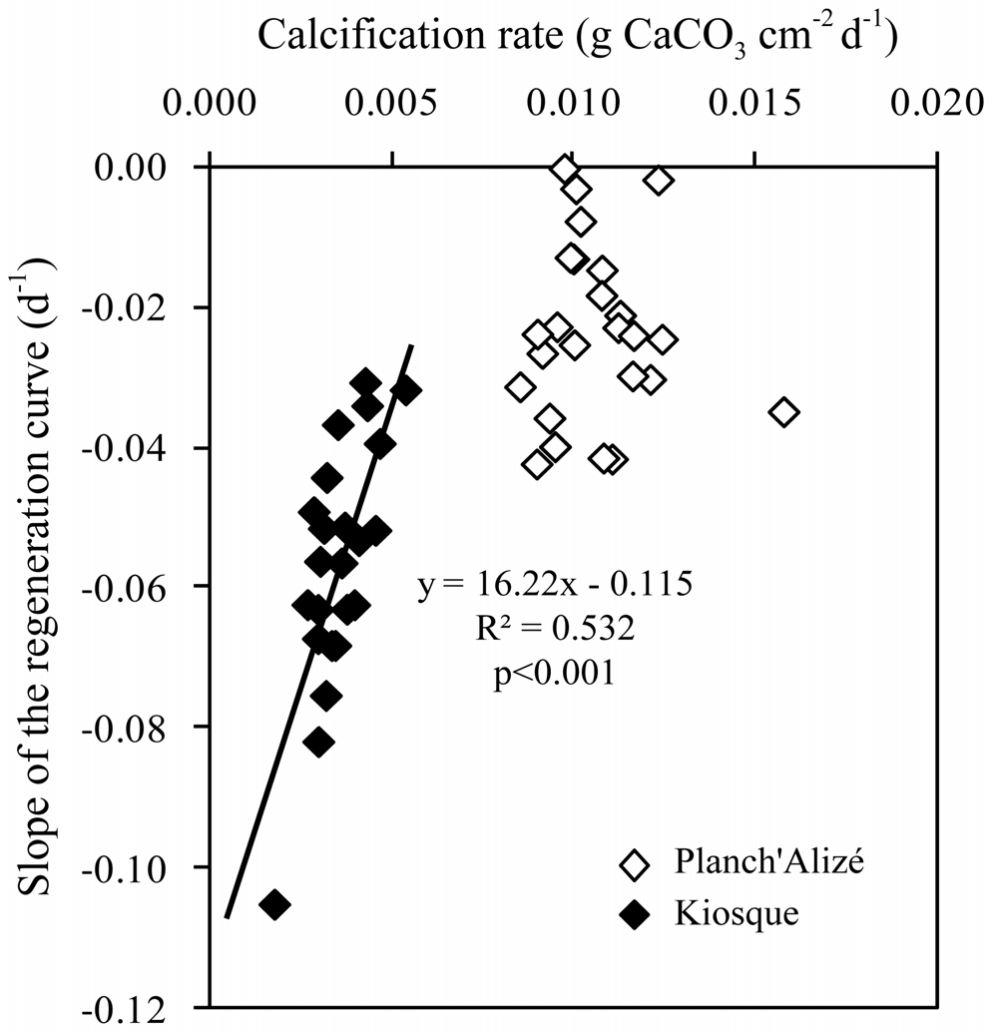
Calcification and lesion healing of *Acropora muricata*. Relationship between calcification rates and lesion healing for injured nubbins from Planch’Alizé and Kiosque.

At Kiosque, the calcification rates of the injured nubbins were lower than that of the controls (*p*<0.001), and rates of calcification and lesion regeneration were negatively correlated (*R^2^* = 0.532, *p<*0.001; Fig. 5). At Planch’Alizé, no difference in growth was observed between injured and control nubbins (*p* = 0.3). At this latter site, lesion regeneration rates were slow and not related to growth (Fig. 5).

*F_v_/F_m_* values of injured and control nubbins were respectively 0.677±0.016 (*n* = 25) and 0.684±0.021 (*n* = 50) at Planch’Alizé, and 0.682±0.020 (*n* = 23) and 0.690±0.016 (*n* = 50) at Kiosque. *F_v_/F_m_* did not significantly differ between sites or treatments (Mann-Whitney U-tests, *p*>0.05), and no significant temporal variation was observed for injured nubbins (Planch’Alizé: *X^2^* = 9.64, *p*>0.05, df = 6; Kiosque: *X^2^* = 6.82, *p*>0.05, df = 6).

## Discussion

While the existence of a trade-off between growth and regeneration was clearly identified for some coral species [17], it is not known whether this relationship varies with environmental conditions. Here, we identified that a specific environment conducive to high zooxanthellae densities in coral tissues may favour growth regardless of damage to the colony.

Environmental conditions at Planch’Alizé, including low water flow, high mean SST and high SST variation, combined with nutrient enrichment from N-rich groundwater seepage [29], likely boost zooxanthellae densities and chlorophyll concentrations [42]. Preponderance of autotrophic energy sources in *A. muricata* at this site is further reflected in the high lipid and low protein content of the nubbins. Indeed, lipids are mainly derived from excess carbon fixed by zooxanthellae [43]. Due to its sheltered location and high irradiance, stressful conditions for corals may also be expected to occur frequently here (Fig. 1a). Lipids represent important energy at reserves during stressful periods [44], and integrated symbionts-host lipogenesis is considered as a photoprotective mechanism during periods of excess irradiance [45].

At Kiosque, the tissue biomass of nubbins tended to be higher than at Planch’Alizé, although the difference was not significant. Higher tissue biomass may reflect the opportunities corals have for heterotrophic feeding (see review in [46]). The exposed site Kiosque, which receives a regular influx of coastal water, provides this advantage and allows corals to maintain vital processes during periods when photosynthetic function is compromised. In an earlier study on the lesion regeneration in massive corals at same sites, the potential importance of heterotrophic feeding at Kiosque reef flat during the summer season [22] was already highlighted.

While the contrasted light and temperature regimes between Planch’Alizé and Kiosque were expected to affect the photosynthetic efficiency in corals, *F_v_/F_m_* values measured in *A. muricata* nubbins did not differ between these sites. Furthermore, lesion infliction did not affect *F_v_/F_m_* values in nubbins, in spite of the stress this likely represents. High *F_v_/F_m_* values were maintained throughout the experiment. Measured photosynthetic efficiencies were higher than those of *A. muricata* nubbins from the Great Barrier Reef kept at a non-stressful temperature [47]. This further suggests that specific environmental conditions at Planch’Alizé did not impair the photosynthesis of *A. muricata* at this site and may not have been stressful for nubbins there. Differences in the photosynthetic efficiency may also be related to differences in zooxanthellae genotypes [48]. However, while in Chagos Archipelago, *A. muricata* is commonly associated with C1-*Symbiodinium* [49], on the Great Barrier Reef, C3-*Symbiodinium* is the predominant subclade present in *Acropora* spp. [50], [51] as identified here.

Homogeneity of lesion sizes avoids an important confounding factor that complicates drawing viable inferences about the regeneration capacity of corals under different environmental conditions [19]. Lesion size in *A. muricata* decreased exponentially over time, which corresponds to the common pattern observed for corals [14], [17], [20]. Despite significantly lower skeletal growth at Kiosque, the mean time required for complete lesion healing was shorter here (81 days) than at Planch’Alizé (192 days). This result is remarkable because it contradicts the general positive relationship found between coral growth and lesion repair ability (see review in [21]). Most studies on lesion regeneration in corals have used massive species (see review in [21]), and only a few studies on *Acropora* spp. are available to compare the slopes of the exponential decay model obtained here. Slopes observed at Planch’Alizé were higher than those of the Caribbean *A. palmata* regenerating similar-sized lesions (−0.040±0.007 [52]), indicating a lower regenerative capacity than that for this flattened branching coral. In contrast, the very steep slopes obtained for *A. muricata* nubbins at Kiosque attest to favourable environmental conditions for coral regeneration at this exposed site.

Vigorous skeletal growth, both in terms of linear extension and calcification, of nubbins from Planch’Alizé was not affected by lesion infliction and may be driven by a high supply of photosynthetic products from zooxanthellae. Indeed, a previous study suggested the stimulating role of photosynthesis on calcification induced by nutrient enrichment at this site [41]. Whereas the lesion regenerations rates at Planch’Alizé were slower and not related to growth, at Kiosque both the reduced skeletal growth of injured lesions compared to controls and the negative relationship between calcification and lesion regeneration rates suggest competition for limited resources between these vital processes. While a high lesion regeneration rate is generally assumed in fast-growing corals [21], our observations suggest that environmental conditions that promote fast skeletal growth may compromise rather than boost the lesion regeneration capacity. This observation may help explain the paradox that while fast growing corals such as *Acropora* spp. are capable of fast tissue regeneration [5], [14], [53], they may nevertheless be prone to substantial partial mortality in their natural environment [54], [55].

Energetic reserves such as lipids are considered to uphold vital life processes [43]. Despite *A. muricata* nubbins from Planch’Alizé having high lipid content, they regenerated lesions slowly. Tissue biomass of nubbins may be a better predictor of the regenerative capacity, as nubbins from Kiosque showed both high tissue biomass and fast regeneration. Essential resources required for lesion regeneration not only involve energy but also interstitial cells, which are shared among different life functions, including reproduction, growth, repair [11], [56] and mucus production. The trade-off between growth and lesion regeneration observed in nubbins from Kiosque complement this view. Very high skeletal extension rates, as observed in nubbins from Planch’Alizé may exert an important drain on interstitial cell availability for tissue repair and may compromise a coral’s regenerative capacity. As proposed by Rinkevich [12], the availability (or depletion) of stem cells will ultimately control observed trade-offs among life-history traits, which are independent of energy availability.

The fact that corals living with high zooxanthellae densities under high irradiation levels require more photosynthetically derived energy to maintain the stability of this symbiosis may provide an alternative explanation for the reduced regeneration capacity of nubbins at Planch’Alizé. While CO_2_-concentrating mechanisms are highly energy consuming, they may play an essential role in preventing CO_2_ limitation of zooxanthellae and its deleterious consequences for both photosystem II and the coral host [57]. Such energetic demands could interfere with lesion regeneration. At Planch’Alizé, where environmental conditions boost zooxanthellae densities alongside with high photosynthetic and calcification rates, our results suggest that growth demands have priority over other life processes. At Kiosque, where zooxanthellae densities, light levels and photosynthetic demands are lower, corals show the expected trade-off between growth and regeneration rates.

Eventually, competition with organisms (such as algae or microbes) that settle inside lesions may hamper lesion regeneration as an energetically costly mechanism [58]. Chronic nutrient enrichment at Planch’Alizé reef flats favours particularly the development of benthic algae, especially during the hot rainy season [27], [59]. We suggest that this chronic disturbance contributed to the seasonal impairment of the lesion regenerative capacity of *P. lutea* [22] and may also have contributed to the slower lesion repair in *A. muricata* at this site during the same hot and warming seasons.

An understanding of key processes of corals is critical to appreciating the divergent population trajectories after exposure to disturbances [60]. Our results suggest that the path to recovery (growth, regeneration, etc.) may strongly differ according to local environmental conditions. Such conditions need to be taken into account when assuming the resilience capacities of coral reefs exposed to increasing levels of disturbances.
